# Simulation and Response Surface Methodology for Predicting Mass Transfer in Coaxial Electrospun Core-Shell Fibers

**DOI:** 10.3390/mi17050606

**Published:** 2026-05-15

**Authors:** Xun Chen, Weiming Shu, Rongguang Zhang, Shize Huang, Xuanzhi Zhang

**Affiliations:** 1 State Key Laboratory of Precision Electronic Manufacturing Technology and Equipment, Guangdong University of Technology, Guangzhou 510006, China; wms2025@126.com (W.S.); zrg941230@163.com (R.Z.); hsz_0914@126.com (S.H.); xuanzhizhang@foxmail.com (X.Z.); 2 School of Electromechnical Engineering, Guangdong University of Technology, Guangzhou 510006, China

**Keywords:** coaxial electrospinning, sensing interface regulation, response surface methodology

## Abstract

Coaxial electrospinning technology enables the fabrication of nanofibers with a core-shell structure, thereby facilitating the encapsulation of functional materials. Its efficacy lies in the precise regulation of mass transfer behavior at the sensing interface. However, achieving the controllable preparation of core-shell fiber structures in complex environments and quantitatively predicting their mass transfer kinetics remain challenging. This study aims to establish a predictive framework combining simulation and experiment. Firstly, finite element simulations using COMSOL clarified that increasing the shell thickness or decreasing its effective diffusion coefficient can significantly delay analyte transport. A model incorporating time-varying parameters further revealed the influence of polymer swelling on the initial release kinetics. Using the diffusion of an aqueous KCl solution as a model system, experiments confirmed that increasing the shell solution concentration is an effective processing strategy for enhancing the mass transfer barrier. Based on the Box-Behnken design and response surface methodology (RSM), a quantitative model linking key process parameters to release kinetic parameters was established. Model diagnostics indicated that the regression equation is significant and reliable. Validation experiments demonstrated that the model possesses good predictive capability for the key release kinetic parameters, with prediction errors within an acceptable range. The framework established in this study indicates that active design of the mass transfer behavior of core-shell fibers can be achieved through process control, providing a quantitative predictive tool and methodological reference for the preparation of controllable mass transfer interfaces for sensing applications.

## 1. Introduction

Chemical and semiconductor sensors serve as core sensing elements in fields such as environmental monitoring, industrial process control, medical diagnosis, and the Internet of Things. Their system performance directly depends on reliability, sensitivity, and long-term stability [1,2,3,4,5]. As detection environments become increasingly complex, non-specific loss, deactivation, or performance degradation of sensing materials—such as metal oxides, conductive polymers, and two-dimensional nanomaterials—during operation has become a bottleneck limiting their practical use [6,7,8]. Traditional methods for immobilizing sensing materials often fail to control their spatial distribution and interfacial microenvironment, leading to issues such as sensor signal drift, poor reproducibility, and shortened service life [9,10,11]. Therefore, developing novel carrier structures that can effectively encapsulate and protect sensing materials while regulating the mass transfer behavior between target analytes and the sensing materials is key to improving overall sensor performance [12].

Coaxial electrospinning technology offers a highly promising platform for constructing such an ideal interface with controlled mass transfer functionality [13,14]. This technique enables the one-step fabrication of continuous nanofibers with a core-shell structure. The core layer can be loaded with various functional sensing materials, while the shell layer can serve both as a protective barrier and a selective mass transfer channel [15,16]. By adjusting parameters such as shell thickness, compactness, chemical properties, and the core-to-shell size ratio, the diffusion rate of target analytes towards the active sites in the core, as well as the intrusion of interferents or harsh environmental components, can be finely regulated. This allows for the active design of sensor response kinetics, selectivity, and stability [17,18]. Recent studies have confirmed that sensors based on coaxial electrospun core-shell fibers demonstrate higher sensitivity and selectivity in detecting various gases, such as ammonia [11], triethylamine [19], and acetone [20], compared to single-material sensors. This enhancement is attributed not only to the formation of heterojunctions but also to the optimized mass transfer achieved through controlled exposure of reactive sites by the shell layer [21].

Despite its promising prospects, achieving the controllable fabrication of core-shell fiber structures—particularly key parameters determining mass transfer resistance, such as shell thickness and compactness—remains a major challenge [22]. Coaxial electrospinning is a complex process involving multi-physics coupling of electrical forces, fluid dynamics, and solvent evaporation. The final fiber morphology is constrained by the interplay of various factors, such as polymer solution properties and process parameters [23,24]. Traditional single-factor, one-at-a-time experimental approaches are unable to fully reveal the influence of these complex interactions on the fiber structure, leading to significant randomness in the manufacturing process. This unpredictability makes it difficult to quantitatively establish a reliable relationship between “process parameters—core-shell structure—mass transfer performance,” and fails to meet the requirement for “on-demand design” of interfacial mass transfer behavior for specific sensing applications [25,26].

Response Surface Methodology (RSM) is an experimental design approach that integrates mathematical modeling, statistical analysis, and optimization, providing a powerful tool for solving such multi-variable optimization problems and establishing quantitative predictive models [27,28]. However, research systematically applying RSM to coaxial electrospinning for precise regulation of its core-shell structure and prediction of its impact on interfacial mass transfer behavior remains insufficient.

Therefore, to address the aforementioned challenges and achieve the predictable design of mass transfer behavior in core-shell fibers, this study proposes a strategy integrating simulation and experimentation. We present a research framework consolidating “simulation-modeling-validation,” aiming to establish a quantitative methodology for predicting and designing the interfacial mass transfer behavior of coaxial electrospun core-shell fibers. This study is contextualized by the design requirement for “controlled mass transfer interfaces” in future high-performance chemical/semiconductor sensors. The core innovation lies in: Firstly, using potassium chloride (KCl) diffusion in water as a model system, COMSOL finite element simulation is employed to mechanistically elucidate the regulatory principles of core-shell structural parameters on mass transfer kinetics. Subsequently, adopting the Box-Behnken experimental design combined with RSM, a quantitative predictive model linking key process parameters to the structural characteristics of core-shell fibers is established within the selected process parameter space. Finally, by experimentally measuring the release kinetics of KCl, the predictive capability of the established model for key mass transfer indicators, such as initial release time and release rate, is validated.

The objective of this work is not to directly present a complete sensor device, but to establish a predictable quantitative “process-structure-mass transfer” relationship model. This model aims to provide a pre-optimization tool for subsequent researchers to design core-shell fiber sensing interfaces with customized mass transfer behavior, thereby contributing to the development of a new generation of highly stable, sensitive, and customizable nanofiber-based sensors.

## 2. Materials and Methods

### 2.1. Materials

Hexafluoroisopropanol (HFIP) was purchased from Shanghai Myriad Chemical Technology Co., Ltd. (Shanghai, China), Polylactic acid (PLA, Mw = 80,000) was supplied by Chengdu Huaxia Chemical Reagent Co., Ltd. (Chengdu, China), Polyvinyl alcohol (PVA, ≥97.5%) was obtained from Kuraray Co., Ltd. (Tokyo, Japan), Potassium chloride (KCl, ≥99.8%) was provided by Guangzhou Shuopu Biotechnology Co., Ltd. (Guangzhou, China), Deionized water was acquired from Guangzhou Rentai Technology Co., Ltd. (Guangzhou, China).

### 2.2. Finite Element Model for Analyte Diffusion 

In the study of chemical and semiconductor sensors, precise regulation of mass transfer behavior at the sensing interface is crucial. In this work, potassium chloride (KCl) was selected as the model substance loaded in the core to simulate the diffusion behavior of typical hydrated ions or small-molecule analytes near the sensing interface. Owing to its chemical stability and ease of quantitative monitoring, KCl serves as an ideal model system for investigating mass transfer processes within nanoconfined spaces. The associated research methodology has been widely applied in fundamental studies of analyte diffusion behavior [29,30]. By investigating the diffusion and release of KCl from the core through the polymer shell, we can quantitatively evaluate the effectiveness of the core-shell structure as a mass transfer barrier. This aligns with the core physical mechanism in many sensors, where the shell is used to control the rate at which analytes or interferents reach the active sites.

To mechanistically understand how core-shell structural parameters and material properties jointly influence mass transfer kinetics, we first constructed an axisymmetric finite element model using COMSOL Multiphysics 6.4. This model simulates the trans-shell diffusion and release process of KCl ions under different core-shell dimensions, diffusion coefficients, and partition coefficients. The primary aim of the simulation is mechanism verification and trend prediction—namely, to elucidate the regulatory principles of key variables such as shell thickness, effective diffusion coefficient, and interfacial partition coefficient on the release kinetics—rather than to precisely replicate all the intricate details of the complex experimental system.

#### 2.2.1. Initial Static Model: Idealized Assumptions and Parameter Settings

**Figure 1:**
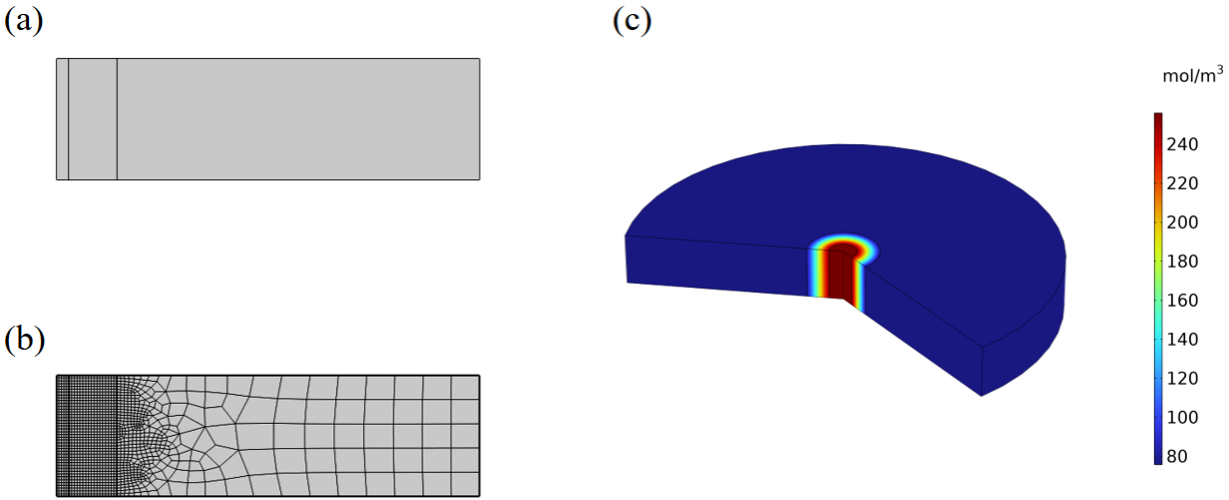
(**a**) Geometric model of coaxial fiber. (**b**) Mesh generation. (**c**) Release diagram.

The initial model was constructed based on the following simplifying assumptions to focus on the core physical mechanisms: (1) the fiber is an ideal concentric cylindrical structure; (2) the shell layer is dense, uniform, and has a smooth surface; and (3) the fiber diameter is uniform. These assumptions enable us to analyze the structure-property relationship within a clear and controllable theoretical framework. Figure 1 shows the two-dimensional axisymmetric geometric model of the coaxial fiber, the mesh generation, and a schematic diagram of KCl release. Based on preliminary research and experimental observations, the model parameters are set as listed in Table 1. Among these, the diffusion coefficients of KCl in the shell (PLA) and core carrier (PVA) refer to the typical ranges for hydrated polymers reported in the literature [31,32]. This model allows for the visualization and quantitative analysis of the spatiotemporal evolution of analyte concentration gradients, thereby elucidating the influence mechanisms of various structural factors on the mass transfer efficiency at the sensing interface under idealized conditions.

#### 2.2.2. Extended Model: Time Varying Parameter Simulation Considering Swelling Dynamics

**Figure A1:**
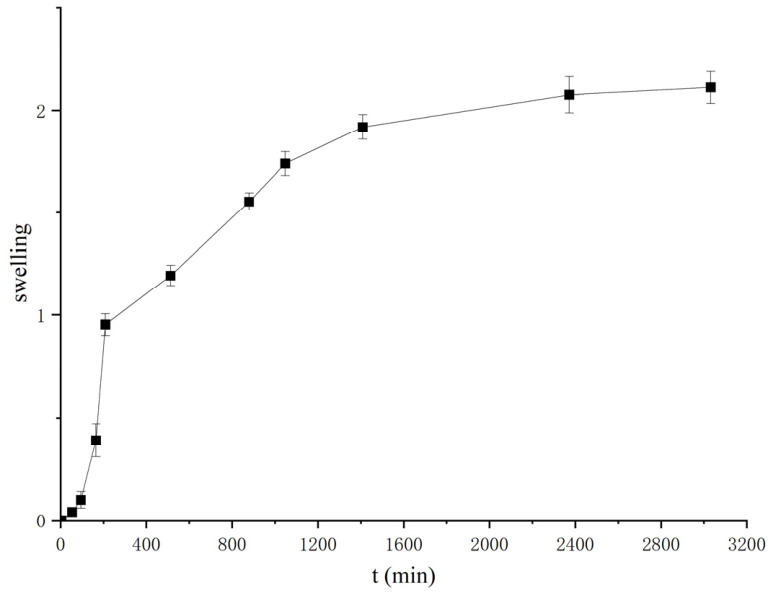
Swelling degree of coaxial fiber.

We acknowledge that the initial static model treats diffusion coefficients and geometric dimensions as fixed values, neglecting the dynamic process in which actual polymer fibers swell in an aqueous environment, leading to time-dependent changes in their size and permeability. To better reflect the physical reality, we have added new simulations considering time-varying parameters. In this extended model, the swelling kinetics of the fiber upon contact with water is incorporated into the model in a simplified form using a piecewise function. During the defined initial swelling stage, the fiber diameter and the effective diffusion coefficient of the shell layer are set as functions that increase over time; after this stage, the parameters stabilize. The parameter settings for these time-varying relationships are partly based on data from our fiber membrane swelling experiments (Figure A1) and partly on the general understanding of diffusion behavior in polymer-water systems. Although the specific functional forms remain semi-empirical, this improvement enables the model to describe the non-linear acceleration phase of the release profile caused by swelling. Consequently, the model trends align more closely with experimental observations and can be used to analyze the contribution of the swelling effect to the overall release kinetics.

#### 2.2.3. Model Limitations Explanation

It is imperative to explicitly state the boundaries and oversimplifications of this simulation work:

Structural Idealization: The model is based on a single, ideally concentric cylindrical fiber. In reality, electrospun fiber mats exhibit diameter distributions, core-shell eccentricity, local defects, and the complex porous structure formed by fiber stacking.

Parameter Uncertainty: The effective diffusion coefficient of the shell layer is a key parameter synthesizing porosity, tortuosity, crystallinity, and polymer-water interactions. The range of literature values used in this model carries a degree of uncertainty.

Scale Discrepancy: The simulation focuses on the intrinsic mass transfer behavior of a single fiber. In contrast, experiments are conducted on membranes composed of tens of fiber layers, which introduce additional mass transfer effects at the membrane level.

Mechanistic Simplification: The model concentrates on diffusion as the physical mass transfer process. It does not incorporate chemical steps that may be involved in actual gas sensing, such as adsorption/desorption, surface reactions, and charge transfer.

The value of this simulation lies in providing a theoretical framework and benchmark for subsequent experimental design and data analysis, which reveals physical mechanisms and predicts trends. By comparing the idealized static model, the time-varying model incorporating swelling, and the experimental data, we can gain deeper insights into the dominant mechanisms behind the observed phenomena and the sources of discrepancies.

### 2.3. Experimental Design and Preparation and Characterization of Coaxial Fiber Membrane

#### 2.3.1. Preparation of Coaxial Fiber Membrane

In this study, coaxial electrospinning technology was employed to fabricate KCl-loaded core-shell fiber membranes. The shell solution was prepared using hexafluoroisopropanol (HFIP) as the solvent and polylactic acid (PLA) as the solute, while the core solution consisted of deionized water as the solvent, with KCl and polyvinyl alcohol (PVA) as solutes. All experiments were conducted under fixed environmental conditions: temperature 22.5 °C, air humidity 45%, and a collection distance of 17 cm. The infusion rates for the core and shell solutions were fixed at 0.05 mL/h and 1.8 mL/h, respectively. This fixed flow rate ratio was selected based on preliminary experiments, aiming to provide the widest adjustable range for subsequent investigations into solution concentration and voltage parameters under the current equipment setup, while ensuring fundamental stability of the jet.

##### Single-Factor Experiment

To preliminarily investigate the influence of process parameters on fiber morphology and KCl release, and to determine the parameter center point and horizontal range for subsequent response surface design, a single factor gradient experiment was first conducted. As shown in Table 2, we sequentially changed the concentration of the shell solution, the concentration of the core solution (PVA), and the applied voltage, with only one factor changed at a time, while fixing the other factors at the central level.

##### Response Surface Experiment

To systematically investigate the interactive effects of process parameters and establish a quantitative predictive model, this study employed a three-factor, three-level Box-Behnken design (BBD). Based on single-factor experiment results, shell solution concentration (A), core solution concentration (B), and working voltage (C) were selected as the design variables. Each factor was set at three coded levels: low (−1), middle (0), and high (1), with specific values detailed in Table 3. This design generated a total of 17 experimental runs. The experimental design matrix and the corresponding combinations of process parameters are presented in Table 4.

#### 2.3.2. Characterization of Core–Shell Fiber Membranes

The core-shell structure of the fibers was directly observed and confirmed using Transmission Electron Microscopy (TEM, LEO 912 Omega, Zeiss, Oberkochen, Germany). For sample preparation, a copper grid coated with a carbon film was placed directly on the collector to collect fibers. From the TEM images of each sample, at least 50 fibers were randomly selected, and their inner diameter (core diameter) and outer diameter (total diameter) were measured using ImageJ 1.54f. The average fiber diameter, shell thickness, and core-to-shell diameter ratio were calculated based on these measurements. This method served as the sole source for the quantitative dimensional data of the core-shell structure in this study. Scanning Electron Microscopy (Hitachi SU8220, Hitachinaka, Ibaraki, Japan) was used for the rapid assessment of the overall morphology, uniformity, and diameter distribution trends of the fiber membranes.

#### 2.3.3. Characterization of Release Kinetics

To investigate the mass transfer behavior of fiber membranes prepared under different process conditions, we systematically measured the release kinetics of KCl. All release experiments were conducted at room temperature. First, the spinning time was controlled to maintain the membrane thickness at 150 ± 8 μm. Prior to release testing, the membrane was immersed in deionized water and stirred for 5 min to remove any solid KCl residue potentially adsorbed on the surface, ensuring that the release originated from the KCl loaded within the internal core. Subsequently, the treated membrane was transferred to a release chamber containing a quantified volume of deionized water. The change in solution conductivity was monitored using a conductivity meter until the value reached a stable plateau, indicating that KCl release was essentially complete. The solution conductivity was converted to KCl concentration using a pre-established calibration curve.

#### 2.3.4. Analysis of KCl Release Curves

To quantify the release kinetics, the Korsmeyer Peppas model was used to analyze the release data. The model equation is as follows:

(1) $$\frac{C_{t}}{C_{\infty }}=K_{KP}t^{n}$$

where $C_{t}$ and $C_{\infty }$ are the cumulative release concentrations at time t and at infinite time, respectively; $K_{KP}$ is the release rate constant; and n is the release exponent, used to indicate the release mechanism. To better fit the release profile with delayed characteristics observed in this study, an initial release time parameter $t_{0}$, determined from experimental data, was introduced. Taking the logarithm of Equation (1) yields the expression used for linear fitting:

(2) $$\ln\frac{C_{t}}{C_{\infty }}=n\ln\left(t-t_{0}\right)+\ln K_{KP}$$

Based on observations of the swelling behavior of the fiber membranes (Appendix A Figure A1), we divided the entire release process into two stages for analysis, using 200 min as the boundary. The release data from 0–200 min and from 200 min to the end of release were separately fitted using Equation (2), yielding two sets of kinetic parameters: $n_{1}$, ${lnK}_{KP1}$ and $n_{2}$, ${lnK}_{KP2}$, as well as the overall characteristic time parameters: initial release time $t_{0}$ and release termination time T. These six parameters will serve as the response variables for the subsequent Response Surface Analysis.

## 3. Results and Discussion

### 3.1. Simulation Analysis of Coaxial Fiber Slow-Release

**Figure 2:**
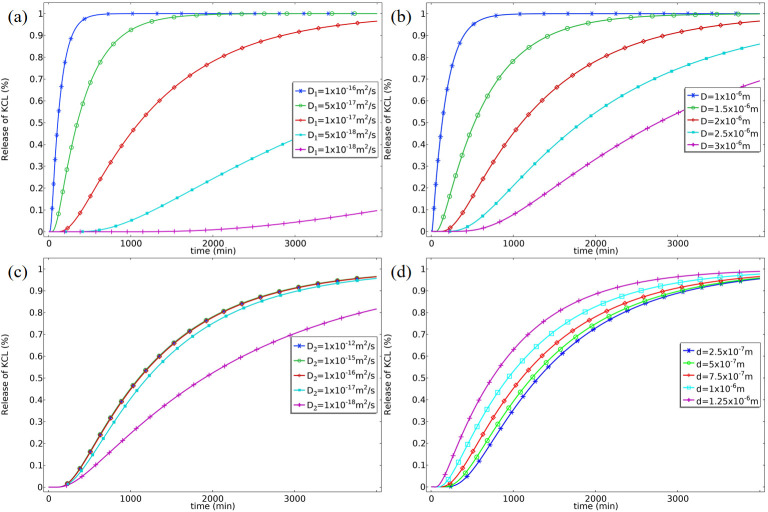
Finite element simulation results of the effects of different structural parameters on the mass transfer behavior of analytes within core-shell fibers. (**a**) Effect of the shell diffusion coefficient on cumulative release; (**b**) effect of shell thickness (represented by the fiber outer diameter) on release kinetics; (**c**) effect of the core diffusion coefficient on cumulative release; (**d**) effect of the core diameter on release kinetics.

COMSOL simulation results reveal, at a mechanistic level, the regulatory principles of core-shell structural parameters and material properties on mass transfer kinetics. As shown in Figure 2, we analyzed the effects of variations in shell diffusion coefficient (D_1_), fiber outer diameter (D, primarily reflecting shell thickness), core diffusion coefficient (D_2_), and fiber inner diameter (d) on the KCl release profile.

Figure 2a,b illustrate the influence of the shell diffusion coefficient (D_1_) and the fiber outer diameter (D), respectively. Simulation results indicate that both decreasing the shell diffusion coefficient and increasing the fiber outer diameter significantly delay the onset of KCl release and slow the overall release rate. This is because both factors directly increase the resistance and path length for analyte diffusion from the core to the external environment. Figure 2c shows that when the core diffusion coefficient (D_2_) is greater than approximately 10–15 m^2^/s, its effect on the release profile is negligible; only below this threshold does a decrease in D_2_ slightly slow the release. This confirms that in systems where core diffusion is relatively fast, the properties of the shell layer are the rate-determining step for the mass transfer process. Figure 2d demonstrates that increasing the core diameter (d) slightly retards the release, primarily due to the increased initial total amount of KCl, which requires a longer time to reach the same release percentage under an identical release flux.

The results from the static model based on fixed parameters clearly validate the core physical mechanism that thickening or densifying the shell layer can effectively retard mass transfer. This provides a theoretical basis for utilizing the coaxial electrospun shell layer to design mass transfer barriers.

To better approximate the real physical system, we extended the initial static model by incorporating a time-varying parameter model that accounts for the swelling kinetics of the fibers. This model gradually couples the time-varying parameters that swelling may induce, including the core/shell dimensions, the diffusion coefficients in the core and shell layers, and the interfacial partition coefficient (K). The simulated system compares the release behavior under four hypothesized conditions: (A) without considering time-varying parameters; (B) considering only the core/shell diameter changes caused by swelling; (C) considering the changes in core/shell diameters and diffusion coefficients due to swelling; and (D) comprehensively considering the changes in core/shell diameters, diffusion coefficients, and the interfacial partition coefficient K caused by swelling.

**Figure 3:**
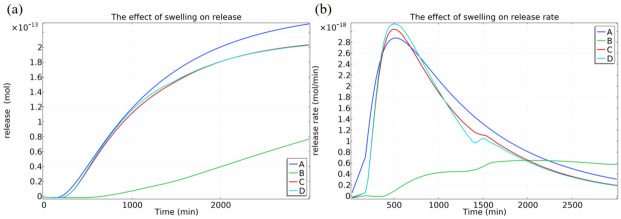
Simulation comparison of the influence of swelling kinetics on the mass transfer behavior of analytes in core-shell fibers. (**a**) Cumulative release curves under different swelling assumptions; (**b**) corresponding release rate curve.

We compared the release behaviors under these four hypothesized conditions (Figure 3). The simulation results considering only the core/shell diameter changes due to swelling (Line B) show a significantly slower release compared to the baseline case without time-variation (Line A), primarily because swelling increases the diffusion path length. However, upon further introducing the changes in diffusion coefficients (Line C) and interfacial partition coefficient K (Line D) induced by swelling, the release behavior becomes more complex. This is because swelling, while increasing fiber volume and path length, also enhances the permeability of the shell layer and alters the interfacial partition equilibrium. Line D, which incorporates all time-varying parameters, shows a cumulative release profile relatively close to Line A, resulting from the counteracting effects of the path length increase and permeability enhancement. The corresponding release rate profile (Figure 3b) further reveals that, under the influence of time-varying parameters, the initial release rate is higher and decays more rapidly. These results collectively indicate that the dynamic swelling process of the polymer shell in the medium is a key factor dominating the early-stage release kinetics. This explains why classical Fickian diffusion models based on fixed parameters struggle to accurately fit the entire experimental release curve.

From the comprehensive simulation results, we draw the following conclusions that provide guidance for experimental design: (1) Increasing the shell thickness through process control or selecting/preparing shell materials with lower permeability is an effective strategy for delaying analyte transport, thereby possibly prolonging sensor response time or enhancing its anti-interference capability. (2) In actual aqueous or humid environments, the swelling behavior of the polymer shell layer is a non-negligible factor, and its dynamic changes dominate the initial mass transfer process.

The mechanisms elucidated by these simulations provide a theoretical basis and explanatory framework for subsequently analyzing how structural differences in the experimentally prepared fiber membranes lead to different KCl release behaviors.

### 3.2. Microscopic Morphology

The overall structure of the coaxial nanofiber membranes was examined using scanning electron microscopy (SEM). The fiber diameter was determined by analyzing transmission electron microscopy (TEM) images with ImageJ software (version 1.54g), whereby approximately 50 nanofibers were randomly selected and measured from each sample.

**Figure 4:**
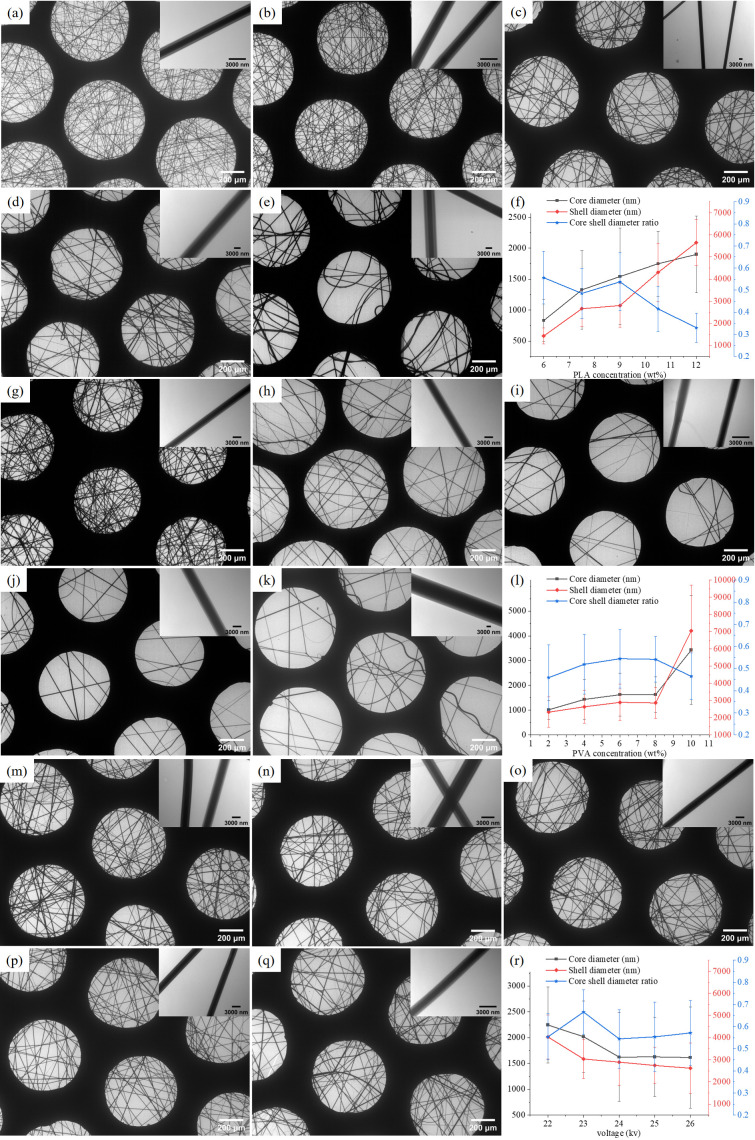
(**a**–**e**) Coaxial fibers and changes in fiber diameter at different concentrations of PLA. (**f**–**l**) Coaxial fibers and changes in fiber diameter under different PVA concentrations. (**m**–**r**) Coaxial fibers and changes in fiber diameter under different voltages.

Figure 4a–f reveals that as the shell solution concentration increases, both the core and shell diameters enlarge, while the core-shell diameter ratio exhibits a declining trend. During the coaxial electrospinning process, the elevation of the shell solution concentration serves as a pivotal factor driving these morphological changes. Primarily, the increased shell concentration leads to a marked rise in its viscosity. In polymer solution electrospinning, solution viscosity is a critical parameter governing jet stability and the ultimate fiber diameter. A shell fluid with higher viscosity possesses a greater resistance to deformation when stretched under the electric field, consequently requiring a larger force to achieve thinning. Under constant electric field force and stretching conditions, this results in the formation of a thicker shell fiber, which aligns with the observed increase in the average outer shell diameter from 1425 nm to 5651 nm. Concurrently, the core inner diameter increased from 830 nm to 1899 nm, primarily as a passive response to the alterations in the shell fluid. Within the coaxial jet, the core fluid is encapsulated and constrained by the shell. When the shell viscosity increases and its flow dominates, the shell becomes difficult to stretch and thin. Simultaneously, the increased shell viscosity enhances the flow resistance exerted on the core, making the encapsulated core polymer more resistant to stretching, thereby leading to a thicker core diameter. The decline in the core-shell diameter ratio can be attributed to the excessively high shell viscosity, which overly strengthens its dominance and control over jet formation, while the influence of the core fluid relatively diminishes. This causes the ultimately formed fiber structure to be predominantly determined by the shell properties, manifesting as a sharp increase in shell thickness and a relative reduction in the core proportion, thus resulting in a decreased core-shell diameter ratio.

Figure 4g–l reveals that as the core solution concentration increases, both the core and shell diameters enlarge, while the core–shell diameter ratio exhibits an initial increase followed by a subsequent decline. Throughout the coaxial electrospinning process, the shell solution concentration and applied voltage were held constant, with the core layer containing 7.5 wt% KCl. The elevated core concentration induces a significant increase in its solution viscosity, which serves as the pivotal physical factor driving these morphological changes. Although the added KCl, acting as an electrolyte, may further enhance the solution conductivity and intensify the jet stretching by the electric field, the results indicate that the change in viscosity is the dominant factor influencing the final fiber morphology. The overall enlargement of both the core and shell diameters can be attributed to the following mechanisms. The increase in core concentration promotes greater molecular chain entanglement, leading to higher solution viscosity. A fluid with higher viscosity possesses greater resistance to deformation during electrical stretching, making it less susceptible to thinning under the same electric field force, ultimately resulting in the formation of a thicker core fiber. Concurrently, the shell diameter increased from 2326 nm to 7045 nm, primarily as a passive response to the increased core viscosity. The enhanced core viscosity strengthens its dragging effect on the shell fluid. Simultaneously, as the core itself becomes more resistant to stretching within the electric field, it consequently impedes the stretching of the encapsulating shell layer. The combined action of these factors leads to the coarsening of the shell layer. The core–shell diameter ratio demonstrates an initial rise to a peak value of 0.545 followed by a decline, reflecting a transition in the dominance between the core and shell layers during jet formation. In the initial stage, the increase in core viscosity from a relatively low level improves the rheological matching between the core and shell fluids. Research indicates that an optimal range for the inner-to-outer layer viscosity ratio exists, which favors the formation of a stable compound Taylor cone and a uniform jet. During this stage, the increased core viscosity enables it to better resist shear and stretching from the shell, allowing for coordinated deformation with the shell layer, thus causing the core–shell diameter ratio to rise. However, when the core concentration continues to increase beyond the optimal range, the excessively high core viscosity strongly hinders its own stretching and thinning process. Under a constant voltage, the fixed electric field force becomes insufficient to effectively draw the core into a finer fiber. Furthermore, the extremely high core viscosity can destabilize the compound jet and impose excessive flow resistance on the shell fluid. Consequently, the core–shell diameter ratio decreases significantly. This phenomenon shares a similar physical mechanism with the case where excessively high shell viscosity leads to a smaller core diameter and increased wall thickness but occurs here under the condition of excessively high core viscosity.

Figure 4m–r demonstrates that during coaxial electrospinning, with constant shell and core solution concentrations and the core layer containing 7.5 wt% potassium chloride (KCl), variations in the applied voltage significantly influence the jet stretching, stability, and solidification processes by altering the intensity of the electric field force. This leads to complex trends in the core inner diameter, shell outer diameter, and the core–shell diameter ratio. The core mechanism lies in the voltage, by modulating the electric field force, markedly affecting the jet’s stretching degree, stability, and solidification rate. The presence of KCl in the core layer as an electrolyte significantly enhances the electrical conductivity of the core solution, rendering the core more sensitive to electric field variations and thereby intensifying these effects. As the voltage increased from 22 kV to 26 kV, the enhanced electric field force dominated the stretching and thinning of the jet. The stronger field force facilitated more sufficient drawing and refinement of the jet, resulting in a decrease in the final solidified fiber diameter. When the voltage was excessively high, the jet exhibited vigorous whipping and unstable motion; however, the powerful electric field stretching force still led to an overall trend of diameter reduction. The core–shell diameter ratio exhibited a trend of initial increase, followed by a decrease, and then a subsequent rise. This complex variation reveals how voltage differentially affects the stretching processes of the core and shell layers, with the high electrical conductivity of the core solution (due to KCl) playing a key role. The ratio increased within the 22–23 kV range. This is because the core solution, possessing higher conductivity due to KCl, responded more intensely to the electric field force and had likely reached a certain threshold before 22 kV. A moderate voltage increase within this range had a lesser effect on the core compared to the shell. The core’s ability to resist stretching or maintain its structure was relatively stronger than the shell’s within this specific voltage window, leading to an increase in the diameter ratio. The voltage ranges of 23–25 kV represented the optimal stretching interval, where the thinning effect of the electric field force was very pronounced. Although the high-conductivity core is sensitive to the electric field, the encapsulation and viscoelastic resistance from the shell solution, coupled with the intense stretching of the overall jet, likely caused sufficient refinement of the core during this stage, resulting in a decrease in the core–shell diameter ratio. At excessively high voltages (beyond 25 kV), jet instability and the “whipping effect” intensified. The vigorous whipping likely caused a differentiation in the stretching forces experienced by the core and shell layers. More importantly, the shell layer might undergo greater stretching under the ultra-high electric field and severe whipping, leading to a relative increase in the proportion of the core layer and thus a rebound in the core–shell diameter ratio.

### 3.3. KCl Release from Coaxial Fiber Membrane

**Figure 5:**
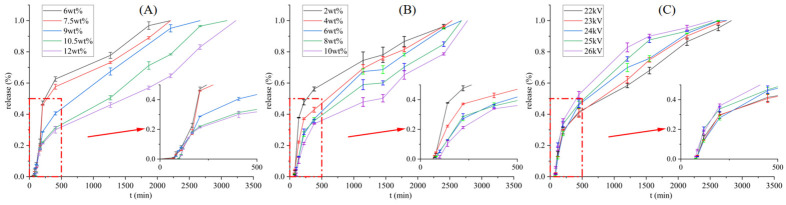
(**A**) KCl release at different shell solution concentrations. (**B**) KCl release at different concentrations of core solution. (**C**) KCl release at different voltages.

To quantitatively evaluate the effectiveness of coaxial fiber membranes prepared under different process conditions as mass transfer barriers, we systematically measured the KCl release kinetics. The experimental results (Figure 5) reveal the intricate influence of process parameters on the release behavior. The overall trend is generally consistent with the simulation predictions in Section 3.1 and the morphology characterization in Section 3.2. However, deviations due to non-ideal structures were also observed, which deepens our understanding of the “process-structure-performance” relationship.

Figure 5A assesses the influence of shell solution concentration on KCl release behavior. The results show that as the shell solution concentration increased from 6 wt% to 10.5 wt%, the fiber diameter and shell thickness increased significantly, accompanied by a marked slowdown in KCl release. This manifested as a longer initial release time and an overall more gradual release profile. This directly validates the mechanism predicted by the simulation that increasing shell thickness can retard mass transfer. However, when the shell concentration was further increased to 12 wt%, its release profile did not show a slower rate within the first 200 min compared to the 9 wt% sample. This anomaly is attributed to the instability of Taylor cone formation and increased jet whipping caused by the excessively high viscosity of the shell solution. This likely induced local misalignment or defects in the core-shell structure, causing parts of the core to be closer to the fiber surface. Consequently, this partially offset the barrier effect provided by the increased shell thickness and even led to an initial burst release. This phenomenon indicates that process optimization must strike a balance between increasing shell thickness and maintaining structural integrity.

Figure 5B evaluates the influence of core solution concentration on KCl release behavior. The results show that an increase in core solution concentration led to a larger core diameter. Theoretically, this should slow the release due to the increased total amount of KCl and slight changes in the diffusion path. The experimental results are largely consistent with this trend, showing a deceleration in release rate as the core concentration increased. The initial increase followed by a decrease in the core-to-shell diameter ratio reflects changes in the rheological compatibility between the core and shell solutions. This may subtly affect the continuity of the core and the uniformity of KCl distribution, thereby contributing to the observed differences in the release profiles.

Figure 5C assesses the effect of voltage on KCl release behavior. The results indicate that higher voltage enhances the stretching force on the jet, leading to finer fibers, which theoretically should accelerate release. Experimentally, fiber membranes prepared at 24–26 kV generally exhibited faster overall release rates compared to those prepared at 22–23 kV. However, for samples prepared at the relatively lower voltages of 22–23 kV, the initial release phase was even slightly faster than that of the 24 kV sample in some cases. This is because the relatively lower electric field force might be insufficient to form a stable and complete shell encapsulation, leading to incomplete coverage of the core material. Consequently, a portion of the KCl is prone to rapid release. This observation further demonstrates that structural defects can significantly undermine the designed efficacy of the mass transfer barrier predicted by theory.

In summary, the KCl release experiments not only successfully quantified the mass transfer delay effect of different core-shell fiber membranes, but also revealed that the variations in release rate, observed anomalies, and kinetic mechanisms could all be qualitatively correlated with structural changes (diameter, thickness, integrity) induced by the process parameters. This thus provides a necessary and reliable experimental data foundation for the subsequent establishment of a quantitative predictive model using the Response Surface Methodology (Section 3.4), and demonstrates the feasibility of achieving controllable mass transfer through process-mediated fiber structure engineering.

### 3.4. Response Surface Analysis and Validation

Based on the 17 sets of experimental data obtained from the Box-Behnken design (Table 5), we performed regression analysis using Design-Expert 11.0 software, aiming to establish quantitative predictive models between the three process parameters and the six release kinetic response variables.

The value of the release exponent $n_{1}$ roughly ranged between 1 and 2, while the value of the release exponent $n_{2}$ roughly ranged between 0.4 and 0.6. According to the Korsmeyer-Peppas model, the release exponent *n* can be used to determine the release mechanism: when *n* ≤ 0.45, release is primarily dominated by Fickian diffusion; when 0.45 < *n* < 0.89, it indicates non-Fickian diffusion; and when *n* ≥ 0.89, it is mainly Case-II transport dominated by swelling or erosion. In this study, all $n_{1}$ values were greater than 0.89, which directly confirms the existence of a significant, non-Fickian transport mechanism dominated by polymer swelling in the initial release stage. The $n_{2}$ values, ranging between 0.45 and 0.60, indicate that the kinetics gradually transition to a diffusion-dominated regime in the later release stage. Swelling experiments also confirmed this. The fitted values for the release rate constants ${lnK}_{KP1}$ and ${lnK}_{KP2}$ were all negative, indicating a relatively slow overall release rate.

#### 3.4.1. Regression Model Establishment and Statistical Analysis

Through fitting the experimental data, second-order polynomial regression equations for the six response variables were obtained, as shown in Equations (3)–(8). To evaluate the significance, goodness-of-fit, and predictive capability of these models, a systematic Analysis of Variance and model diagnostics were performed.

(3) $$t_{0}=-2499.23+55.87A-19.3B+196.7C-0.42AB-2.83AC+1.13BC+1.43A^{2}+0.065B^{2}-3.78C^{2}$$

(4) $$T=5472.25+455.67A+375.06B-516.63C-6.67AB+4AC-0.375BC-12.17A^{2}-20.78B^{2}+7.88C^{2}$$

(5) $$n_{1}=-3.03-4.82A+1.13B+1.94C-0.05AB-0.01AC-0.02BC+0.27A^{2}-0.01B^{2}-0.03C^{2}$$

(6) $$n_{2}=16.74+0.08A+0.24B-1.42C-0.001AB+0.005AC-0.01BC-0.007A^{2}+0.001B^{2}+0.03C^{2}$$

(7) $${lnK}_{KP1}=76.69+22.11A-5.65B/wt\%-14.32C+0.29AB+0.09AC+0.1BC-1.32A^{2}+0.03B^{2}+0.25C^{2}$$

(8) $${lnK}_{KP2}=-128.68-0.54A-1.59B+10.82-0.003AB-0.04AC+0.07BC+0.06A^{2}-0.01B^{2}-0.22C^{2}$$

The corrected Analysis of Variance table (Table 6) clearly presents the F-values and *p*-values for each model, as well as their linear, interaction, and quadratic terms. Among them, the shell solution concentration (A) exhibited the most significant influence on most response variables (particularly $t_{0}$ and T), as indicated by the largest F-value and a *p*-value < 0.0001. This is consistent with the single-factor experiment results, confirming that adjusting the concentration of the shell material is the most effective processing strategy for controlling mass transfer delay. The influence of the core solution concentration (B) was relatively more complex, primarily manifested in its significant impact on the later-stage diffusion parameters ($n_{2}$ and ${lnK}_{KP2}$). The applied voltage (C) also had a significant effect on $t_{0}$, T, $n_{2}$, and ${lnK}_{KP2}$.

The model diagnostic results (Table 7) indicate that the regression equations possess sound statistical reliability. The coefficient of determination (R^2^) for each model is at a high level. Furthermore, their adjusted R^2^ and predicted R^2^ values are close, with a reasonable difference, indicating a good model fit without significant overfitting. The signal-to-noise ratios are all greater than 4, demonstrating that the models have adequate signal discrimination capability.

**Figure 6:**
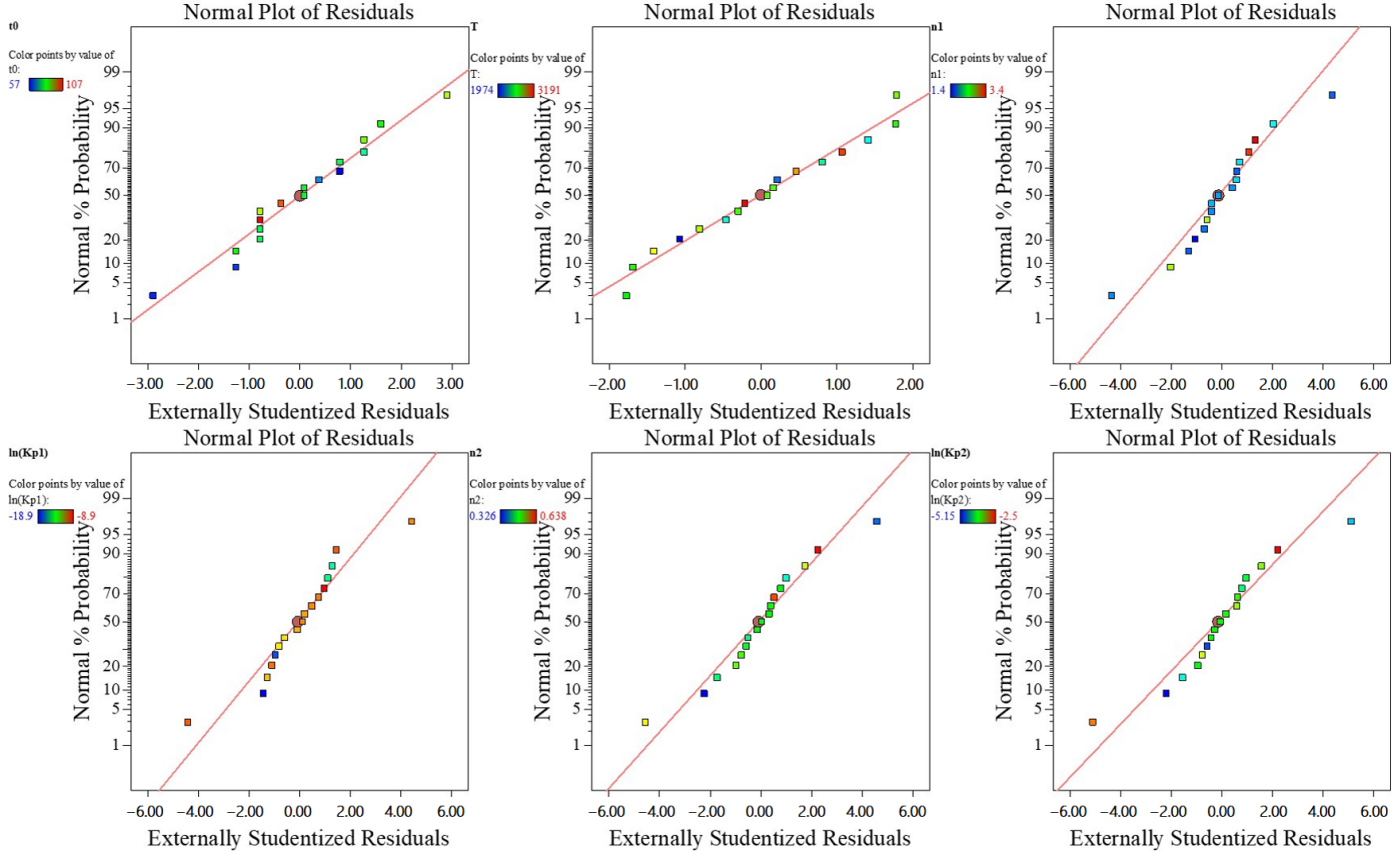
Release residual of parameters.

The normal probability plot of residuals verifies the model’s reliability by assessing whether the data follows a linear distribution. In the normal probability plot shown in Figure 6, the horizontal axis represents the theoretical quantiles of the standard normal distribution, while the vertical axis represents the cumulative probability of the data points under the assumption of a standard normal distribution. The data points in Figure 6 align closely along a straight line, indicating that the residuals conform to a normal distribution, thereby confirming the accuracy of the model.

#### 3.4.2. Model Validation

To evaluate the predictive capability of the established models, three sets of process parameter combinations within and at the boundaries of the experimental design space, which were not used for model building, were selected for validation experiments. Table 8 compares the model-predicted values with the experimentally measured values.

For the two key mass transfer delay indicators—the initial release time ($t_{0}$) and the total release time (T)—the prediction deviations for all three validation points were less than 5%. The prediction deviations for the other four response values were also largely kept within 10%. The average prediction errors for all six response variables were within an acceptable range.

The validation results demonstrate that, within the process parameter space defined in this study, the established RSM models exhibit good predictive capability and guiding value for the KCl release kinetics of the coaxial fiber membranes. It must be noted that the validity boundary of the models is limited by the factors and levels investigated in the experiments. For instance, if fundamental changes occur in process parameters (such as the flow rate ratio) or the material system, the models would require recalibration. However, the core principles revealed by this model, such as the dominance of shell concentration on mass transfer delay, and the demonstrated “Simulation-RSM-Validation” research framework provide a quantitative tool and methodological reference for optimizing the coaxial electrospinning process towards specific mass transfer performance goals.

## 4. Conclusions

This study investigated and validated an integrated “process-structure-performance” framework for predicting and regulating the mass transfer behavior of coaxial electrospun core-shell fiber membranes.

First, finite element simulation mechanistically clarified that shell thickness and effective diffusion coefficient are the key structural parameters regulating analyte transport rate. An extended model incorporating time-varying parameters further revealed that the swelling kinetics of the polymer shell in an aqueous environment significantly influences the initial release behavior, leading to Super Case II transport characteristics. These simulation results provided a theoretical baseline for interpreting experimental phenomena and defined the objectives for structural regulation.

Second, experimental research systematically revealed the influence of process parameters on fiber structure and mass transfer performance. The study demonstrated that increasing the shell (PLA) solution concentration is an effective processing strategy for enhancing the mass transfer barrier, primarily attributable to its significant effect on increasing fiber diameter and shell thickness. Concurrently, the study also observed that when process parameters deviate from the optimal window, structural defects arising from Taylor cone instability or incomplete encapsulation can weaken the intended barrier effect. Release kinetic analysis confirmed a distinct swelling-dominated stage during the initial release period, which later transitioned to a diffusion-dominated regime.

Finally, the response surface models established based on the Box-Behnken design successfully quantified the relationships between key parameters and release kinetic indicators within the selected process parameter space. The developed regression models are statistically significant and demonstrate a good fit. Validation experiments revealed that the prediction errors of the models for the initial release time and the total release time are less than 5%, and the prediction errors for the other four response values are also maintained within 10%.

In summary, the simulation-RSM-validation framework established in this study indicates that actively designing the mass transfer behavior of core-shell fibers can be achieved by regulating coaxial electrospinning process parameters. Although this work utilized an aqueous KCl system as a model, the principles and methodology revealed can provide a general strategic reference and a process starting point for designing core-shell fiber interfaces with customized mass transfer functions, particularly in the development of high-performance sensors for complex environments.

## Figures and Tables

**Table 1:** Model parameter settings.

Parameter	Value
Fiber outer diameter (D)	2 × 10^−6^ m, 3 × 10^−6^ m, 4 × 10^−6^ m, 5 × 10^−6^ m, 6 × 10^−6^ m
Fiber inner diameter (d)	0.5 × 10^−6^ m, 1 × 10^−6^ m, 1.5 × 10^−6^ m, 2 × 10^−6^ m, 2.5 × 10^−6^ m
Initial concentration of KCl (c_0_)	2 × 10^4^ mol/m^3^
Diffusion coefficient of KCl in water (D_0_)	1 × 10^−9^ m^2^/s
Diffusion coefficient of KCl in PLA (D_1_)	1 × 10^−18^ m^2^/s–1 × 10^−16^ m^2^/s
Diffusion coefficient of KCl in PVA (D_2_)	1 × 10^−12^ m^2^/s, 1 × 10^−15^ m^2^/s, 1 × 10^−16^ m^2^/s, 1 × 10^−17^ m^2^/s, 1 × 10^−18^ m^2^/s

**Table 2:** Single factor gradient experiment.

Core Flow Rates 0.05 mL/h; Shell Flow Rate: 1.8 mL/h; Collection Distance 17 cm; Temperature 22.5 °C; Air Humidity 45%; Concentration of KCl 7.5 wt%			
Gradient Experiment	Concentration of Shell Solution (wt%)	Concentration of Core Solution (wt%)	Voltage (kV)
Shell concentration gradient experiment	6, 7.5, 9, 10.5, 12	6	24
Core concentration gradient experiment	9	2, 4, 6, 8, 10	24
Voltage gradient experiment	9	6	22, 23, 24, 25, 26

**Table 3:** Coding levels of electrospinning process parameters.

Design Variable	Denotations	Factor Level		
		−1	0	1
Concentration of shell solution (wt%)	A	7.5	9	10.5
Concentration of core solution (wt%)	B	4	6	8
Voltage (kV)	C	23	24	25

**Table 4:** Experimental schemes.

Experimental Coding	Concentration of Shell Solution (wt%)	Concentration of Core Solution (wt%)	Voltage (kV)
1	9	6	24
2	9	8	25
3	7.5	6	25
4	9	6	24
5	9	4	23
6	9	4	25
7	7.5	8	24
8	9	6	24
9	9	6	24
10	10.5	6	23
11	9	8	23
12	10.5	4	24
13	10.5	8	24
14	10.5	6	25
15	7.5	6	23
16	9	6	24
17	7.5	4	24

**Table 5:** Experimental result.

Experimental Coding	Shell Solution Concentration A (wt%)	Core Solution Concentration B (wt%)	Working Voltage C (kV)	Release Start Time$\;t_{0}$ (min)	Release End Time T (min)	$n_{1}$	${lnK}_{KP1}$	$n_{2}$	${lnK}_{KP2}$
1	9	6	24	78	2626	1.86	−11.23	0.47	−3.71
2	9	8	25	83	2580	1.68	−10.30	0.48	−3.87
3	7.5	6	25	64	2112	3.30	−18.07	0.33	−2.50
4	9	6	24	80	2683	1.64	−10.10	0.48	−3.77
5	9	4	23	74	2622	1.61	−9.80	0.50	−3.98
6	9	4	25	60	2400	1.76	−10.66	0.44	−3.44
7	7.5	8	24	78	2286	3.40	−18.90	0.42	−3.28
8	9	6	24	78	2666	1.72	−10.49	0.48	−3.85
9	9	6	24	80	2730	1.68	−10.28	0.48	−3.80
10	10.5	6	23	103	3191	1.40	−8.90	0.64	−5.15
11	9	8	23	88	2805	1.70	−10.25	0.62	−4.95
12	10.5	4	24	91	2887	1.60	−9.76	0.51	−4.14
13	10.5	8	24	107	3119	1.60	−10.00	0.56	−4.63
14	10.5	6	25	91	3007	1.90	−10.81	0.55	−4.41
15	7.5	6	23	57	2320	2.73	−15.60	0.45	−3.48
16	9	6	24	83	2680	1.84	−11.09	0.49	−3.83
17	7.5	4	24	57	1974	2.75	−15.22	0.36	−2.82

**Table 6:** Analysis of Variance and Significance Test of Release Parameter Regression Equation.

Source of Variance	Model	A	B	C	AB	AC	BC	A^2^	B^2^	C^2^
F-Statistic ($t_{0}$)	58.35	367.09	111.95	13.82	1.02	11.82	3.31	7.16	0.0349	9.81
*p*-Value ($t_{0}$)	<0.0001	<0.0001	<0.0001	0.0075	0.3457	0.0109	0.1116	0.0317	0.8572	0.0165
F-Statistic (T)	136.14	1068.63	71.27	60.99	1.11	0.0998	0.0016	2.19	20.17	0.181
*p*-Value (T)	<0.0001	<0.0001	<0.0001	0.0001	0.3273	0.7613	0.9696	0.1827	0.0028	0.6833
F-Statistic ($n_{1}$)	22.91	138.9	1.88	6.18	3.63	0.0386	0.2545	55.32	0.1161	0.1535
*p*-Value ($n_{1}$)	0.0002	<0.0001	0.2122	0.0418	0.0983	0.8498	0.6294	0.0001	0.7433	0.7068
F-Statistic (${lnK}_{KP1}$)	26.11	161.36	3.24	5.63	4.76	0.1262	0.264	59.51	0.1133	0.4383
*p*-Value (${lnK}_{KP1}$)	0.0001	<0.0001	0.1151	0.0494	0.0654	0.7329	0.6232	0.0001	0.7463	0.5291
F-Statistic ($n_{2}$)	36.16	208.69	27.89	68.48	0.0663	0.7868	4.48	4.25	0.1005	11.36
*p*-Value ($n_{2}$)	<0.0001	<0.0001	0.0011	<0.0001	0.8042	0.4045	0.072	0.0783	0.7604	0.0119
F-Statistic (${lnK}_{KP2}$)	40.06	240.44	33.99	68.67	0.0111	0.7091	3.59	3.23	0.5235	9.83
*p*-Value (${lnK}_{KP2}$)	<0.0001	<0.0001	0.0006	<0.0001	0.9191	0.4276	0.1	0.1155	0.4928	0.0165

**Table 7:** Goodness-of-Fit and Diagnostic Metrics for the Response Variable Regression Models.

	R^2^	Adjusted R^2^	Predicted R^2^	Adeq Precision
$t_{0}$ (min)	0.9868	0.9699	0.8641	27.4192
T (min)	0.9943	0.9870	0.9544	41.2347
$n_{1}$	0.9769	0.9471	0.6519	19.5046
${lnK}_{KP1}$	0.9711	0.9339	0.6322	15.6876
$n_{2}$	0.9789	0.9519	0.6872	21.0974
${lnK}_{KP2}$	0.9810	0.9565	0.7186	21.9357

**Table 8:** Predicted values, actual values, and deviation of KCl release.

		Shell: 8 wt% Core: 7 wt% Voltage: 24 kV	Shell: 10 wt% Core: 5 wt% Voltage: 25 kV
$t_{0}$	predict	75.16	94.71
	measurement	79	97
	deviation	4.9%	2.4%
$n_{1}$	predict	2.16094	1.75868
	measurement	2.02	1.63
	deviation	6.98%	7.32%
${lnK}_{KP1}$	predict	−14.8181	−10.0117
	measurement	−14.13	−10.88
	deviation	4.85%	−8.87%
$n_{2}$	predict	0.429038	0.507404
	measurement	0.44	0.53
	deviation	2.56%	4.45%
${lnK}_{KP2}$	predict	−3.37266	−4.02833
	measurement	−3.21	−4.2
	deviation	4.82%	4.26%
T	predict	2414.74	2930.53
	measurement	2506	3060
	deviation	3.6%	4.2%

## Data Availability

The data that support the findings of this research are available from the corresponding author upon reasonable request.

## Appendix A

Figure A1 shows the mass change of coaxial fibers immersed in water over time, where the mass change is used to represent the degree of expansion of the fiber membrane. The calculation formula is as follows:

$$swelling=\frac{W_{0}-W_{t}}{W_{0}}$$

Within 0–200 min, the membrane mass increased to 2 times its original mass, whereas from 200–3000 min, it only increased to approximately 3.2 times the original mass. This indicates that an extremely intense swelling phenomenon occurred in the fiber membrane during the initial stage.
